# Osteoporosis and Periodontal Diseases: Exploring Shared Pathways, Bidirectional Links, and Integrated Management in a Narrative Review

**DOI:** 10.7759/cureus.97943

**Published:** 2025-11-27

**Authors:** Naif Alwithanani

**Affiliations:** 1 Department of Oral and Maxillofacial Surgery and Diagnostic Science, College of Dentistry, Taif University, Taif, SAU

**Keywords:** association, bidirectional, osteoporosis, periodontitis, review

## Abstract

Osteoporosis and periodontal diseases are chronic conditions characterized by bone loss, affecting millions of people globally, particularly in aging populations. This narrative review synthesizes evidence of their epidemiological associations, shared pathophysiological mechanisms, diagnostic correlations, and therapeutic intersections to elucidate bidirectional relationships and inform clinical practice. A comprehensive literature search was conducted across PubMed/MEDLINE, Scopus, Web of Science, Embase, and Cochrane Library from inception to September 2025, with no date restrictions. Search terms included “osteoporosis,” “bone mineral density,” “postmenopausal bone loss,” combined with “periodontal disease,” “periodontitis,” “alveolar bone loss,” and “attachment loss,” using Boolean operators. Medical Subject Headings and Emtree terms were applied where relevant. Additional keywords targeted mechanistic overlap, including “receptor activator of nuclear factor kappa-B ligand,” “osteoprotegerin,” “interleukin-6,” and “tumor necrosis factor-alpha.” The search prioritized epidemiological studies, systematic reviews, meta-analyses, clinical trials, and basic scientific reports. Supplementary sources included hand-searching reference lists, Google Scholar, and conference abstracts from the International Association for Dental Research, European Federation of Periodontology, and American Society for Bone and Mineral Research. Inclusion was limited to English-language peer-reviewed articles addressing epidemiological, biological, diagnostic, or therapeutic links. Non-human studies, case reports, and editorials were excluded. Two reviewers independently screened the titles and abstracts, with consensus resolving discrepancies. Evidence reveals consistent associations between low systemic bone mineral density and increased periodontal attachment loss, particularly in postmenopausal women, mediated by the receptor activator of nuclear factor kappa-B ligand/osteoprotegerin (RANKL/OPG) axis and proinflammatory cytokines. Shared risk factors such as aging, smoking, diabetes, and estrogen deficiency amplify susceptibility. Antiresorptive therapies may preserve alveolar bone, whereas periodontal treatment reduces systemic inflammation. Clinical implications include cross-screening and multidisciplinary care to mitigate the risks of fracture and tooth loss. Limitations include study heterogeneity and reliance on a cross-sectional design. Future research should prioritize longitudinal studies, biomarker development, and integrated care models to advance the precise management of these interconnected diseases.

## Introduction and background

Osteoporosis and periodontal diseases are two prevalent chronic conditions that disproportionately affect aging populations, sharing potential bidirectional links through systemic inflammation, bone remodeling pathways, and shared risk factors such as age, smoking, and hormonal changes [1]. Osteoporosis is characterized by reduced bone mineral density (BMD) and microarchitectural deterioration of bone tissue, leading to an increased fracture risk, with a global prevalence affecting over 200 million individuals, predominantly postmenopausal women [2]. Periodontal diseases, including gingivitis and periodontitis, involve inflammatory destruction of tooth-supporting structures driven by dysbiotic oral biofilms, resulting in alveolar bone loss and potential tooth mobility, affecting nearly 47% of adults over 30 years worldwide [3].

Emerging evidence suggests mechanistic overlaps, including the role of receptor activator of nuclear factor kappa-B ligand (RANKL) in both systemic skeletal resorption and localized alveolar bone destruction as well as the influence of proinflammatory cytokines such as interleukin-6 (IL-6) and tumor necrosis factor-alpha (TNF-α) [4]. Postmenopausal estrogen deficiency exacerbates both conditions by upregulating osteoclast activity, whereas conditions such as diabetes and glucocorticoid use act as common modifiers [5]. Epidemiological studies have reported associations between low BMD and increased periodontal attachment loss, and meta-analyses have indicated higher odds of periodontitis in osteoporotic individuals [6]. Conversely, severe periodontitis may contribute to systemic bone loss via bacteremia-induced inflammation, although causality remains debatable.

Despite these insights, discrepancies exist in clinical outcomes, diagnostic criteria, and therapeutic implications, necessitating a consolidated synthesis. This narrative review aimed to critically appraise the epidemiological, pathophysiological, and clinical interconnections between osteoporosis and periodontal diseases, highlighting evidence gaps and potential integrative management strategies to inform future research and multidisciplinary care.

## Review

Search methodology

A comprehensive literature search was conducted to identify studies examining the interconnections between osteoporosis and periodontal diseases. The search encompassed five electronic databases, including PubMed/MEDLINE, Scopus, Web of Science, Embase, and Cochrane Library, from their inception through September 2025, with no date restrictions to capture both foundational and emerging evidence. The search strategy combined core terms using Boolean operators: ("osteoporosis" OR "bone mineral density" OR "BMD" OR "postmenopausal bone loss") AND ("periodontal disease*" OR "periodontitis" OR "gingivitis" OR "alveolar bone loss" OR "attachment loss"), supplemented by Medical Subject Headings (MeSH)/Emtree terms such as "Osteoporosis," "Osteoporosis, Postmenopausal," "Bone Density," "Periodontitis," and "Alveolar Bone Loss." Additional keywords targeting mechanistic overlap included "RANKL," "osteoprotegerin," "OPG," "IL-6," "TNF-alpha," and "inflammation."

The search prioritized epidemiological studies (cross-sectional, cohort, and case-control), systematic reviews, meta-analyses, clinical trials, and basic science reports, whereas narrative reviews and expert opinions were considered for contextual insights. Supplementary sources included hand-searching reference lists of included articles, screening the first 200 results in Google Scholar, and reviewing conference abstracts from the International Association for Dental Research (IADR), European Federation of Periodontology (EFP), and American Society for Bone and Mineral Research (ASBMR), with targeted searches in high-impact journals such as the Journal of Clinical Periodontology, Osteoporosis International, and Journal of Bone and Mineral Research. Inclusion was restricted to English-language, peer-reviewed articles reporting original data or synthesized evidence on epidemiological associations, biological mechanisms, diagnostic correlations, or therapeutic implications. Non-human studies, case reports, editorials, and studies addressing only one condition without linkage to another were excluded. Title and abstract screening was performed independently by two reviewers, and discrepancies were resolved by consensus, followed by full-text retrieval of eligible studies. The search was updated iteratively during manuscript preparation to incorporate the newly published evidence. Consistent with the narrative review methodology, no formal quality appraisal tools were applied; however, the study design, sample size, and adjustment for confounders were critically evaluated during evidence synthesis to ensure a balanced, clinically relevant narrative.

Epidemiology and risk factor overlap

Epidemiological data consistently demonstrate a higher prevalence of periodontal disease among individuals with osteoporosis, particularly postmenopausal women [7]. Cross-sectional studies from the National Health and Nutrition Examination Survey (NHANES) revealed that menopausal women were significantly associated with osteoporosis and had 1.66-fold greater odds of moderate-to-severe periodontitis [8]. Similarly, a large Korean cohort (n = 22,948) found that osteoporotic women exhibited significantly greater clinical attachment loss (CAL ≥ 4 mm) and probing depths compared to age-matched controls with normal BMD, and 1.25 times higher risk of periodontal diseases [9]. Meta-analyses corroborate these findings, reporting pooled odds ratios of 1.6-2.1 for periodontitis in osteoporotic versus non-osteoporotic populations [6,8,9].

Shared risk factors amplified this association. Aging induces senescent changes in immune responses and bone remodeling, increasing the susceptibility to both conditions. Smoking, a potent inhibitor of osteoblast function and periodontal healing, increases the risk independently and synergistically. Cigarette smoking adversely affects the differentiation of mesenchymal stem cells and osteoblasts during osteogenesis while simultaneously facilitating osteoclast differentiation in the context of bone resorption [10]. Diabetes mellitus, through advanced glycation end-products and microvascular compromise, promotes alveolar bone resorption and systemic osteoporosis [11].

Postmenopausal estrogen decline is a pivotal link; estrogen inhibits RANKL expression and osteoclastogenesis, and its deficiency accelerates skeletal and alveolar bone loss [9]. Glucocorticoid therapy, rheumatoid arthritis, and low body mass index (BMI) further compound the risk, creating a multifactorial web of susceptibility [11,12]. Notably, while most studies have focused on postmenopausal women, emerging data suggest similar associations in elderly men, particularly those with hypogonadism or vitamin D deficiency [13]. However, longitudinal studies tracking the incidence of osteoporosis in patients with periodontitis remain scarce, limiting causal inference.

Pathophysiological mechanisms

Biological interplay between osteoporosis and periodontitis centers on shared bone resorption pathways. Both conditions involve dysregulated osteoclast activity, mediated by the RANKL/osteoprotegerin (OPG) axis [1]. In periodontitis, periodontal pathogens such as *Porphyromonas gingivalis* stimulate gingival fibroblasts and immune cells to upregulate RANKL and promote localized alveolar bone destruction [14]. Systemically, chronic low-grade inflammation from periodontitis elevates circulating RANKL and proinflammatory cytokines (IL-1β, IL-6, and TNF-α), which may exacerbate skeletal resorption in osteoporosis-prone individuals [6]. Conversely, systemic osteoporosis may reduce alveolar bone density, rendering periodontal tissues more vulnerable to inflammatory insult [15].

**Figure 1:**
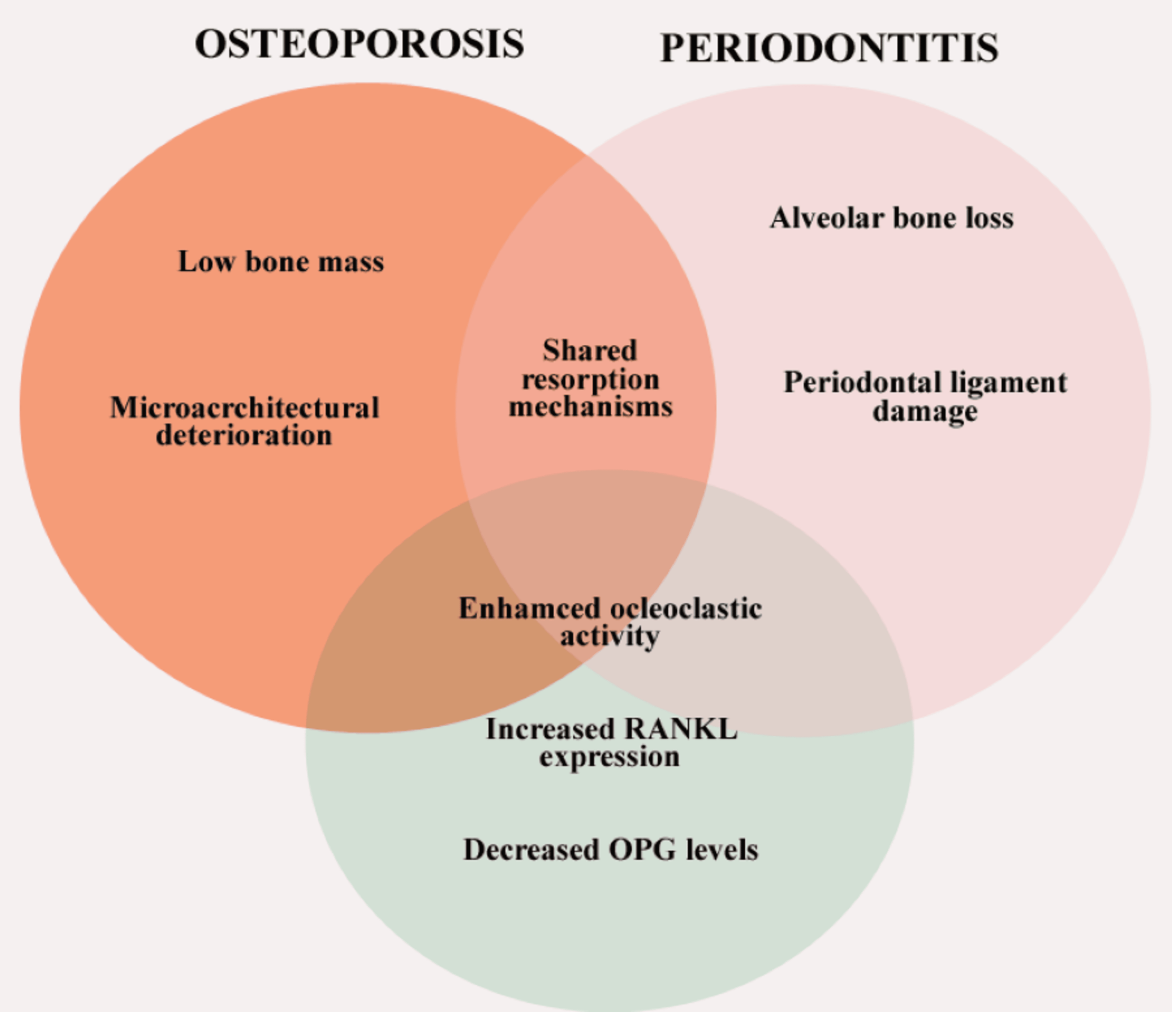
Mechanism of pathophysiology. RANKL: receptor activator of nuclear factor kappa-B ligand; OPG: osteoprotegerin. Graphical image is designed using Canva (Canva Pty Ltd, Sydney, Australia; www.canva.com). Image credit: Naif Alwithanani.

Estrogen deficiency is the central driver. In ovariectomized rats, accelerated alveolar bone loss mirrors femoral trabecular deterioration, which is reversed by estrogen replacement [16]. Proinflammatory cytokines from periodontal pockets enter systemic circulation via ulcerated epithelium, activating hepatic acute-phase responses and inhibiting OPG, thus tilting the RANKL/OPG ratio toward resorption [17]. Microbiome studies have revealed that severe periodontitis alters gut microbiota, potentially influencing systemic bone homeostasis via the gut-bone axis [18,19]. Genetic polymorphisms in the vitamin D receptor, IL-6, and RANKL genes have been implicated in dual susceptibility [15]. While mechanistic convergence is compelling, human studies have rarely quantified circulating RANKL or cytokine levels in dual-disease cohorts, leaving direct translational evidence incomplete. The mechanism of pathogenesis is depicted in Figure 1.

Diagnostic correlations

Clinical and radiographic correlations between systemic BMD and periodontal status are well documented, but variable. Dual-energy X-ray absorptiometry (DXA)-measured femoral and lumbar BMD negatively correlates with radiographic alveolar bone loss and CAL in postmenopausal women [20]. Cone-beam computed tomography (CBCT) revealed reduced trabecular density in the jaws of osteoporotic patients, even in the absence of periodontitis [21]. Kalinowski et al. [22] reported that the Fracture Risk Assessment (FRAX) BMI tool, along with radiographic assessment of periodontitis, can be used to predict osteoporotic fractures in women. Payne et al. [23] evaluated 58 periodontal patients experiencing menopause who were enrolled in a maintenance program. Among these patients, 41 exhibited normal BMD, whereas 17 were classified as osteoporotic. The study reported an increased incidence of alveolar bone loss, along with a reduction in both crestal and subcrestal density, in women with osteoporosis and estrogen deficiency.

However, diagnostic discordance was observed. Some studies have reported no association between BMD and periodontal parameters, possibly because of site-specific bone turnover rates or confounding by local factors such as occlusal trauma [24]. Esfahanian et al. [25] conducted a review to report the association between periodontitis and osteoporosis and concluded that while 11 studies supported an association, six studies did not. Kribbs [26] investigated the correlation between periodontal disease and osteoporosis. They conducted a comparative analysis of the mandibular bone mass in 85 women diagnosed with osteoporosis and 27 women with normal bone density, revealing that the osteoporotic cohort exhibited reduced mandibular bone mass and density, as well as diminished cortical thickness at the gonion, in contrast to the normal cohort. No significant discrepancies in clinical periodontal measurements were observed between the osteoporotic and normal populations.

Elders et al. [27] conducted a comparative analysis of the clinical parameters associated with periodontitis and the height of the alveolar bone in relation to BMD of the lumbar and metacarpal bones. The investigation revealed no statistically significant differences in the occurrence of gingival bleeding, probing pocket depths, gingival recession, and marginal bone levels between subjects exhibiting low BMD and those demonstrating high BMD, suggesting that inflammatory burden, not bone density per se, drives clinical severity. Overall, while BMD screening in patients with severe periodontitis and periodontal evaluation in osteoporotic cohorts may enhance risk stratification, standardized protocols are lacking.

Therapeutic intersections

A therapeutic overlap offers opportunities for integrated management. Bisphosphonates, a first-line treatment for osteoporosis, inhibit osteoclast activity and may preserve alveolar bone in periodontitis. Randomized trials have shown that alendronate reduced CAL progression and radiographic bone loss in postmenopausal women under both conditions [28]. However, osteonecrosis of the jaw risk, although low (<1:10,000 patient-years in oral bisphosphonate users), necessitates pretreatment periodontal sanitation [29]. Denosumab, a RANKL inhibitor, prevents lipopolysaccharide (LPS)-induced osteoclast formation, thus stabilizing alveolar bone in mice, whereas zoledronate inhibits bone destruction but not osteoclast formation [30].

**Figure 2:**
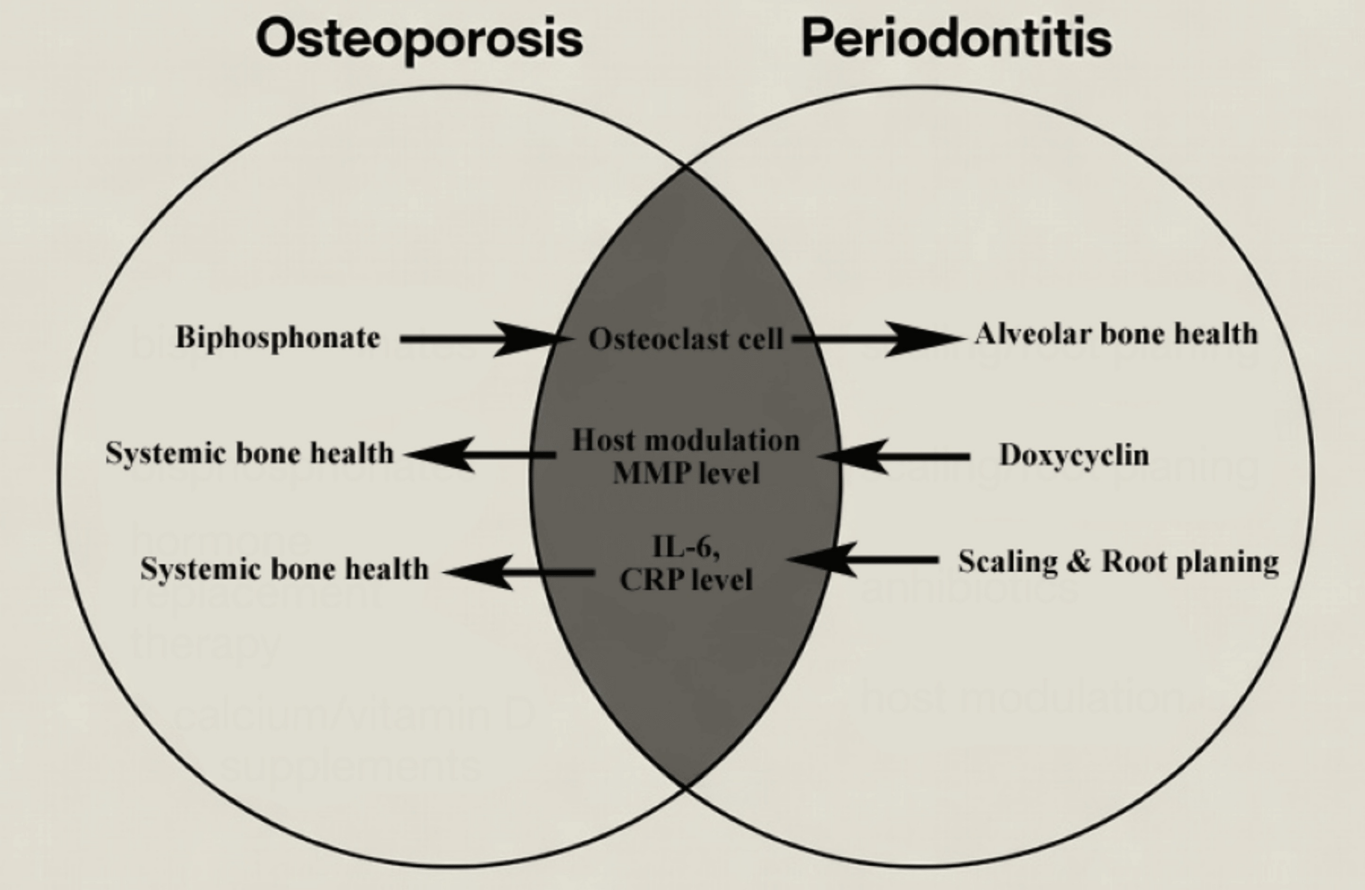
Therapeutic intersection. MMP: matrix metalloproteins; IL-6: interleukin-6; CRP: C-reactive protein. Graphical image is designed using Canva (Canva Pty Ltd, Sydney, Australia; www.canva.com). Image credit: Naif Alwithanani.

Periodontal therapy may improve systemic bone health. Non-surgical scaling and root planing reduce serum IL-6 and C-reactive protein (CRP) levels, potentially attenuating systemic inflammation [31]. Adjunctive host modulation with subantimicrobial-dose doxycycline inhibits matrix metalloproteinases in both periodontal and osteoporotic contexts [32]. Statins, used for dyslipidemia, exhibit pleiotropic anti-inflammatory and bone-anabolic effects and improve periodontal outcomes [33]. Sirtuin 1 (Sirt 1) activator resveratrol has been found to promote alveolar bone formation and prevent alveolar bone loss in mice; however, it has been clinically validated in humans [34]. Emerging therapies targeting the microbiome (such as probiotics and bacteriocins) can effectively reduce alveolar bone loss by modifying the gut microbiota [35]. Lifestyle interventions, such as smoking cessation, weight-bearing exercise, and calcium/vitamin D supplementation, can also benefit both conditions synergistically (Figure 2).

Clinical implications

The bidirectional relationship between osteoporosis and periodontal disease has significant clinical implications in multidisciplinary care. Dentists should consider osteoporosis screening in postmenopausal women with severe periodontitis, particularly in those with multiple tooth loss or radiographic alveolar rarefaction. Conversely, endocrinologists and rheumatologists managing osteoporosis should inquire about oral health and refer patients to periodontal evaluation, especially in patients receiving antiresorptive therapy. Pretreatment dental clearance is essential before initiating bisphosphonates or denosumab to minimize the risk of osteoradionecrosis of the jaw. Integrated care models, such as those combining periodontal maintenance with osteoporosis monitoring, may improve treatment adherence and outcomes. Patient education that emphasizes shared risk factors (smoking and poor nutrition) can empower preventive strategies. In dental implant planning, osteoporotic patients require careful assessment of the jawbone quality, potentially benefiting from adjunctive bone-sparing therapies. Ultimately, recognizing these conditions as interconnected manifestations of dysregulated bone metabolism encourages a holistic approach that reduces morbidity from fractures and tooth loss.

Future recommendations

Future research should prioritize longitudinal cohort studies to establish temporality and causality between periodontitis and incident osteoporosis. Biomarker panels integrating salivary, serum, and gingival crevicular fluid analytes could enable non-invasive dual-risk prediction. Randomized controlled trials evaluating the impact of periodontal intervention on BMD trajectories, and vice versa, are needed. Advanced imaging (such as high-resolution peripheral quantitative CT) should characterize the jawbone microarchitecture in osteoporotic cohorts. Microbiome-bone axis studies using multiomics approaches may uncover novel therapeutic targets. Development of clinical guidelines for cross-screening and co-management in dental and medical societies is warranted. Finally, health economic analyses that assess the cost-effectiveness of integrated care models will support policy implementation.

Limitations

This narrative review is limited by its non-systematic nature, which introduces a potential selection bias despite broad search strategies. Heterogeneity in the diagnostic criteria for periodontitis and osteoporosis across studies complicates this synthesis. Most evidence is derived from cross-sectional designs, precluding causal inferences. Confounding by shared risk factors (age, smoking, and diabetes) has been incompletely adjusted for in many studies. Generalizability is restricted by the under-representation of men, non-Caucasian populations, and premenopausal women. Finally, the rapidly evolving therapeutic landscape (such as romosozumab and novel biologics) outpaces current evidence of periodontal interactions. A summary of the review is given in Table 1.

**Table 1:** Summary of epidemiological, mechanistic, diagnostic, and therapeutic evidence supporting the bidirectional relationship between osteoporosis and periodontal diseases. BMD: bone mineral density; RANKL: receptor activator of nuclear factor kappa-B ligand; OPG: osteoprotegerin; ONJ: osteonecrosis of jaw; OR: osteoradionecrosis; NHANES: National Health and Nutrition Examination Survey; BMI: body mass index; CBCT: cone-beam computed tomography.

Domain	Key findings	Representative studies/evidence	Clinical/research implications
Epidemiology	Higher prevalence of periodontitis in osteoporotic individuals, particularly postmenopausal women; pooled OR ≈ 1.6–2.1.	NHANES data [8]; Lee 2022 [9]; Martínez-Maestre et al. 2010 [6]	Screening for periodontal disease in osteoporotic patients and vice versa.
Shared risk factors	Age, smoking, diabetes, estrogen deficiency, glucocorticoid use, rheumatoid arthritis, low BMI.	Lerner 2006 [13]; Păunică et al. 2023 [11]; Rodríguez-Lozano 2019 [12]	Lifestyle modification and systemic control can mitigate both diseases.
Pathophysiology	Common RANK/RANKL/OPG signaling; inflammatory cytokines (IL-1β, IL-6, and TNF-α); estrogen deficiency accelerates both skeletal and alveolar bone loss.	Hofbauer and Heufelder 2001 [4]; Kajiya et al. 2010 [14]; Martínez-García 2025 [17]	Anti-RANKL or anticytokine strategies may have dual benefit.
Microbiome-bone axis	Periodontitis-induced dysbiosis affects gut microbiota, influencing systemic bone metabolism.	Sulaiman et al. 2024 [18]; Sjögren et al. 2012 [19]	Future therapies may target oral-gut microbiome modulation.
Diagnostic correlations	Reduced systemic BMD correlates with alveolar bone loss; CBCT shows jawbone demineralization; some studies show no link.	Tezal et al. 2000 [20]; Kanneppady et al. 2025 [21]; Kribbs 1990 [26]; Elders et al. 1992 [27]	Dual assessment (BMD + periodontal status) improves risk stratification.
Therapeutic intersections	Bisphosphonates and denosumab reduce alveolar loss; periodontal therapy reduces systemic inflammation; adjuncts like statins and resveratrol beneficial.	Rocha et al. 2004 [28]; Kuritani et al. 2018 [30]; Mohan et al. 2014 [31]; Tahamtan et al. 2020 [33]	Integrated dental-medical management optimizes outcomes; monitor for ONJ.
Future directions	Need for longitudinal, multiomics, and interventional studies; biomarker validation and guideline development.	Narrative synthesis	Cross-disciplinary precision management models recommended.

## Conclusions

Osteoporosis and periodontal diseases share epidemiological, pathophysiological, and therapeutic intersections, which are rooted in dysregulated bone remodeling and systemic inflammation. Postmenopausal women exhibit heightened susceptibility with shared risk factors that amplify dual morbidity. While antiresorptive therapies stabilize alveolar bone and periodontal interventions may attenuate systemic inflammation, evidence gaps persist in causality, diagnostics, and long-term outcomes. Multidisciplinary collaboration, such as integrating dental and medical care, offers synergistic benefits and reduces fracture and tooth loss risks. Future longitudinal studies, biomarker validation, and guideline development are essential to translate mechanistic insights into precise prevention and management strategies for these interconnected chronic conditions.
